# Mini Review of Reliable Fabrication of Electrode under Stretching for Supercapacitor Application

**DOI:** 10.3390/mi13091470

**Published:** 2022-09-05

**Authors:** Haeji Kim, Paolo Matteini, Byungil Hwang

**Affiliations:** 1 School of Integrative Engineering, Chung-Ang University, Seoul 06974, Korea; 2 Institute of Applied Physics “Nello Carrara”, National Research Council, Via Madonna del Piano 10, 50019 Sesto Fiorentino, Italy

**Keywords:** stretchable supercapacitors, multiwall carbon nanotubes, conducting polymers, deformation, wearable electronics

## Abstract

Currently, there is an increasing demand for portable and wearable electronics. This has necessitated the development of stretchable energy storage devices, while simultaneously maintaining performance. Hence, the electrodes and electrolyte materials used in stretchable supercapacitors should be robust under severe mechanical deformation. Polymers are widely used in the fabrication of stretchable supercapacitors. It is not only crucial to choose good polymer candidates with inherent advantages, but it is also important to design suitable polymer materials for both electrodes and electrolytes. This mini-review explains the concept of stretchable supercapacitors, the theoretical background of polymer-based electrodes for supercapacitors, and the fabrication strategies of stretchable electrodes for supercapacitors. Finally, we present the drawbacks and areas that still need to be developed.

## 1. Introduction

A supercapacitor has a much higher capacitance value than those of general capacitors [1,2,3,4,5,6]. However, unlike other capacitors, supercapacitors have lower voltage limits, which can compensate the performance gradients between rechargeable batteries and typical electrolytic capacitors [7,8,9,10,11,12,13,14,15,16,17]. A typical capacitor consists of two electrodes, where the gaps are filled with a dielectric material [18]. Under an applied voltage, the electrodes pull electrons, and the electrical charge is stored in the space between the electrodes. Simultaneously, the dielectric material placed between the two electrodes becomes polarized, which helps increase the capacitance. Supercapacitors are similar to typical capacitors except that they use an electrolytic solution as the wedging material instead of a dielectric material [19]. When a voltage is applied, an “electric double layer” is created, which produces positive and negative charges along the boundaries of the electrodes and electrolytic solution. The electrical charge is accumulated in this double layer. To increase the charge accumulation, it is important to increase the porosity of the electrodes, in fact, porous electrodes have a large effective surface area. Activated carbon is the most widely used for the electrode because of its high porosity. 

Energy storage density of supercapacitors is 10–100 times larger than those of electrolytic capacitors; they shows ultra-fast charge and discharge speed [20]. These advantages make supercapacitors promising candidates for energy storage applications. Supercapacitors differ from batteries in several ways. In batteries, chemical reactions occur between the electrolytic solution and electrodes [21]. However, in supercapacitors, electrons move between the two electrodes [22]. Batteries are mostly used with a specific volume and weight, and they have a high energy density. Meanwhile, supercapacitors have a high power density; hence, they can be operated at low and high temperatures. 

Since the first patent in 1957 [23], supercapacitors have been widely used in various fields, such as electric motilities [24], hybridized vehicles [25], and supplementary energy systems [26]. As mentioned previously, supercapacitors use porous conductors, such as active carbon [27], carbon nanotubes (CNTs) [28], and graphene flakes [29] as electrode materials. Owing to their porosity, these electronic conductors provide large interfacial areas between the electric double layers. However, the brittleness of these conductors limits their stretchability. Therefore, there have been intensive studies to develop stretchable electrodes for supercapacitors [30]. Different types of stretchable supercapacitors have been reported using novel designs, such as serpentines [31], kirigamis [32], meshes [33], and corrugated structures [34]. However, these structures are fabricated using brittle materials, which causes fracture when combined with stress concentration. The stretchability can be improved by adding elastomers [35]; however, the insulating nature of elastomers adversely affects the performance of the supercapacitors [36].

## 2. Fundamentals of Supercapacitors

**Figure 1:**
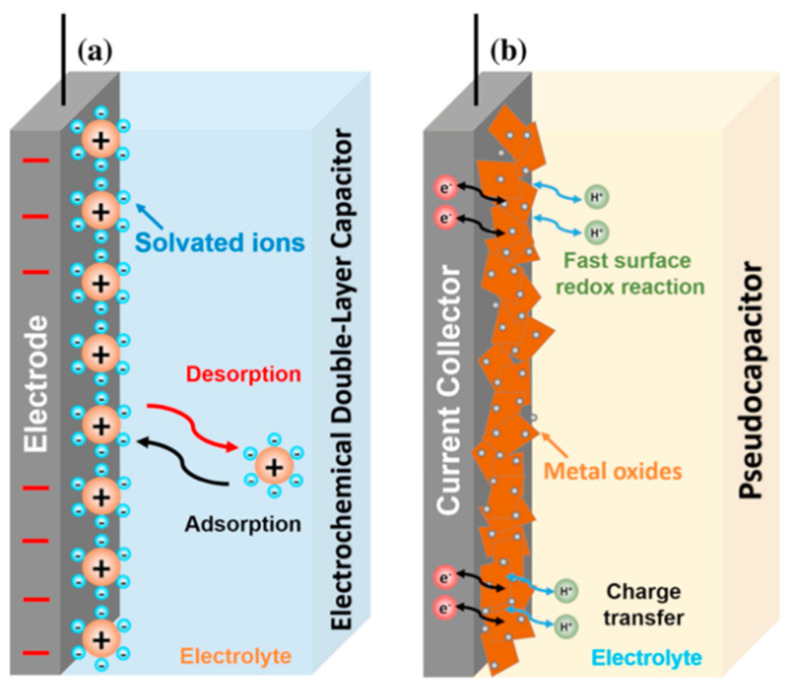
Comparison of charging in (**a**) EDLC (carbon) and (**b**) pseudocapacitor (metal oxides) [37]. Reproduced with permission from ref. [37].

Materials for electrodes of supercapacitors are categorized into electric double-layer capacitor (EDLC) types (Figure 1a) and pseudocapacitive types (Figure 1b) [37]. The EDLC type includes carbon-based materials, such as active carbon, template carbons, CNTs, and graphene [37]. The pseudocapacitive type mostly includes metal oxides, nitrides, sulfides, conducting polymers (CPs), and polymer composites [37]. EDLCs store energy via electrostatic accumulation of charge at the interface between the electrodes and the electrolyte. On the other hand, pseudocapacitors store energy by redox reactions at the surface; thus, the energy density and specific capacitance of pseudocapacitors are much higher than those of the EDLC type.

**Figure 2:**
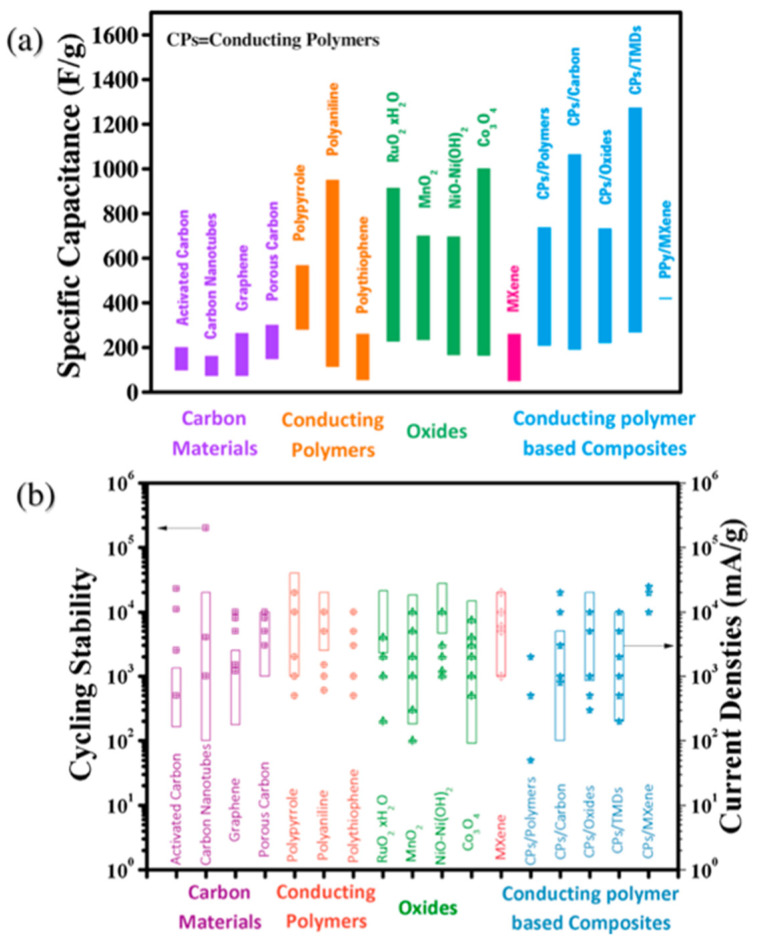
Comparison of (**a**) specific capacitance and (**b**) cycling stability and current density range for typical electrode materials for supercapacitor applications [37]. Reproduced with permission from ref. [37].

Figure 2a shows the specific capacitance values of various active materials used in supercapacitors. The specific capacitance of most carbon-based materials (EDLC type) is less than 300 F/g [38]. Conductive polymers, such as polyaniline, polypyrrole, and polythiophene, generally exhibit higher capacitance values than those of carbon-based materials [39]. Among the CPs, polyaniline has a higher energy storage capability than polypyrrole and polythiophene [40,41]. For pseudocapacitive types, transition metal oxides show high specific capacitances owing to their complex electronic states, various crystalline phase structures, and tunable nanostructures [37]. Therefore, supercapacitors fabricated using transition metal oxides show specific capacitance values higher than ~300 F/g. Hybrid electrodes consisting of CPs and other inorganic electrode materials exhibit even higher capacitance values [39]. Figure 2b shows the cycling stability and current density range of typical active materials used in supercapacitors. The supercapacitors using a single active material or CP-based composites show a similar life cycle, 10^3^–10^4^ cycles. However, activated carbon and CNT electrodes exhibit large variations in the cyclic life and current density range for charge/discharge, while porous carbon and graphene show relatively smaller variations. 

## 3. Stretchable Supercapacitors

### 3.1. Hydrogel-Based Supercapacitors

**Figure 3:**
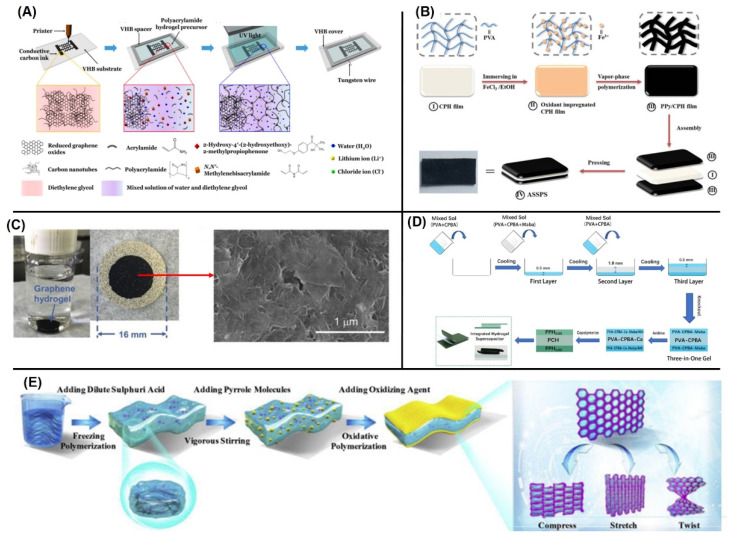
Fabrication process of (**A**) a stretchable supercapacitor by coating ink on a hydrogel elastomer [44], (**B**) PPy-CPH hydrogel-based supercapacitor [45], (**C**) graphene-hydrogel using phenylenediamine-based supercapacitor [46], (**D**) demonstration of a self-healable supercapacitor using all-hydrogel materials [47] and (**E**) all-in-on hydrogel supercapacitor [48]. Reproduced with permission from ref. [44,45,46,47,48].

**Figure 4:**
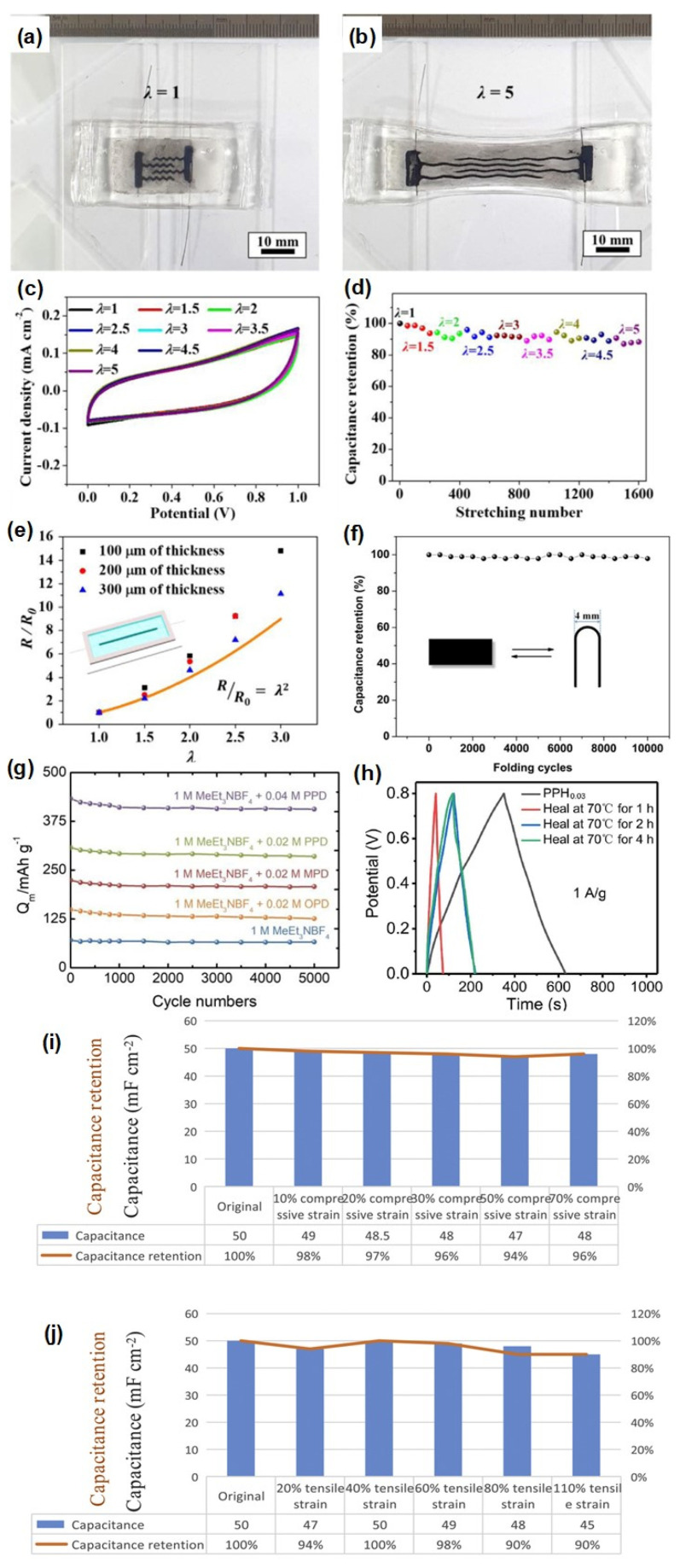
Effect of retention of diethylene glycol in the supercapacitor after printing. Photographs of the supercapacitor in (**a**) unstretched state and (**b**) stretched state (λ = 5). (**c**) CV profiles of the supercapacitor at different stretching amplitudes. (**d**) Capacitance retention of the supercapacitor after different stretching cycles. (**e**) Change in the resistance (R/R_0_) of the printed electrode as a function of the uniaxial stretching amplitude. The inset shows the schematic of the sample [44]. (**f**) Change in capacitance of PPy-CPH hydrogel-based supercapacitor as a function of folding cycles [45]. (**g**) Change in capacitance of graphene hydrogel using phenylenediamine-based supercapacitor [46]. (**h**) Change in electric potential of a self-healable supercapacitor using all-hydrogel materials [47]. Change in capacitance of all-in-on hydrogel supercapacitor under (**i**) compressive and (**j**) tensile strain [48]. Reproduced with permission from ref. [44,45,46,47,48].

Hydrogels, which are polymer networks containing water, are widely used as ionic conductors in many stretchable devices [42]. Hydrogels exhibit excellent ionic conductivity, biocompatibility, toughness and stretchability [43]. Carbon particles are added to hydrogels to form stretchable electronic conductors, which remarkably increase the performance of supercapacitors [37]. Song et al. introduced technology for fabricating stretchable supercapacitors by coating ink on a hydrogel elastomer [44]. Supercapacitors can be fabricated (Figure 3A) with an ink consisting of mixture of graphene flakes and carbon nanotubes [44]. A binary solvent consisting of water and diethylene glycol permeates the percolating network of electrodes penetrated in the polymer matrix [44]. The high stretchability of the supercapacitor results from the interpenetrating networks. Without a polymer network, the printed supercapacitor shows low stretchability with the breakage of the percolating carbon network under small tensile strain. On the other hand, the larger strain value under repeated tensile strain can be achieved by interpenetrating the percolating carbon network with a polymer chain. Sliding of the graphene flakes to CNTs accommodates the imposed strain without losing their electrical connection significantly. The entrapped carbon particles in the polymer networks are due to the bigger dimensions of the carbon particles compared to the mesh size of the polymer chain [44]. The percolating carbon/polymer network and the tungsten electrodes are reliable under repeated tensile deformation. Furthermore, the surface area of the carbon particles is not influenced by the deformation due to their high stiffness. This makes the supercapacitor stretchable and independent of stress [44]. The fabricated electrode does not show any visible damage after uniaxially stretching to 5 times the initial dimensions; moreover, it shows high reversibility (Figure 4a,b). The cyclic voltammetry (CV) curves of the supercapacitor obtained at different stretching amplitudes are identical, which shows that the capacitance of the supercapacitor is independent of mechanical stretch. Cyclic stretching was applied at stretching amplitudes of 1.5, 2, 2.5, 3, 3.5, 4, 4.5, and 5 for a total of 200 cycles. The supercapacitors retained 88.3% of their initial capacitance after 1600 cycles (Figure 4c,d). The failure of the percolating network of carbons was analyzed by measuring the resistance change of the electrode under stretching (Figure 4e). The results show that the resistance increases with stretching due to tensile deformation [44].

There were more reports on hydrogel-based supercapacitors. For example, in the work by Zang et al., conducting polymer-based hydrogel composites was prepared by using a polypyrrole (PPy) and poly(vinyl alcohol)-H_2_SO_4_ hydrogel (CPH) hydrogel [45]. As shown in Figure 3B, the composite hydrogels were fabricated through a vapor-phase polymerization of PPy interpenetrating a cross-linked CPH film. The fabricated composites showed a stretchability of ~377%. The capacitance was retained even after 10,000 cycles of folding tests (Figure 4f). Fang et al. also reported a hydrogel-based supercapacitor where a graphene hydrogel using phenylenediamine was utilized for electrolyte as shown in Figure 3C [46]. They showed a high stability by retaining 98% of capacitance after 5000 cycles of charge-discharge tests (Figure 4g). Zou et al. demonstrated a self-healable supercapacitor using all-hydrogel materials [47]. A sandwich structure consisting of the polyaniline (PANI)-polyvinyl alcohol (PVA) hydrogel electrodes (Figure 3D) provided a high stretchability of 633%. The fabricated supercapacitor can restore the electrochemical performance by more than half of the initial values after a damage-healing process (Figure 4h). Furthermore, Yin et al. reported a deformable all-in-one hydrogel supercapacitor by using a conducting polymer [48]. The polypyrrole-polyvinyl alcohol/dilute sulphuric acid-polypyrrole (PHP) sandwiched device (Figure 3E) had 110% of stretchability while retaining > 90% of initial capacitance at 110% of compressive and tensile strains (Figure 4i,j).

### 3.2. Rubber Composite-Based Supercapacitors 

Stretchable supercapacitors can also be used in wearable and portable devices because they can withstand large deformations and unexpected tensile strain without significantly losing their electronic performance [49]. The main challenge in achieving high-performance stretchable supercapacitors is the accurate tuning of the material composition and structure for fabrication of stretchable electrodes. Currently, there are various methods for developing stretchable electrodes and devices, but the following two strategies are commonly used: (1) structural design for modification of non-stretchable materials into wavy, cellular, mesh, helical, spring-like, honeycomb-like, and pyramid forms, which allows the material to endure large deformations or applied stress [49], (2) integration of non-stretchable parts on stretchable substrates, such as polyurethane, poly(dimethylsiloxane), polyurethane acrylate, styrene/ethylene/butylene-styrene, and gel electrolyte [50]. The first method is very complex and expensive, and the fabricated material exhibits a low strain value of ~50% [49]. In the second method, the poor electrical conductivity of the elastic substrates and dense packing of active materials lead to low specific capacitance and capability rate of the stretchable supercapacitor [49]. This review discusses a strategy for fabricating stretchable electrodes with high stretchability and excellent electrochemical performance. 

**Figure 5:**
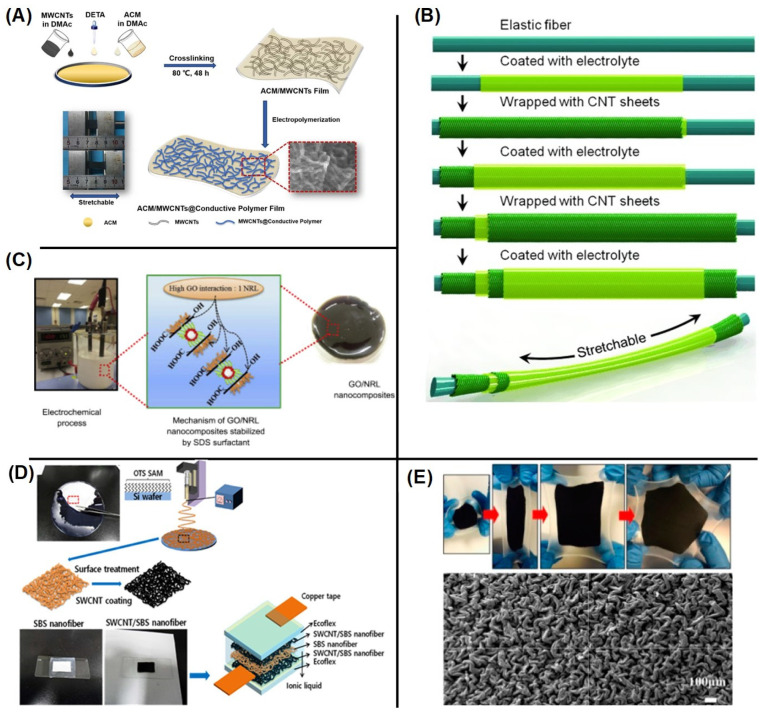
Fabrication process of (**A**) stretchable ACM/MWCNTs@CP film electrodes [49], (**B**) stretchable, fiber supercapacitor [51], (**C**) graphene oxide/natural rubber latex nanocomposites [52], (**D**) CNT-deposited rubber nanofiber electrode-based supercapacitor [53] and (**E**) isotropic buckled CNT films [54]. Reproduced with permission from ref. [49,51,52,53,54].

**Figure 6:**
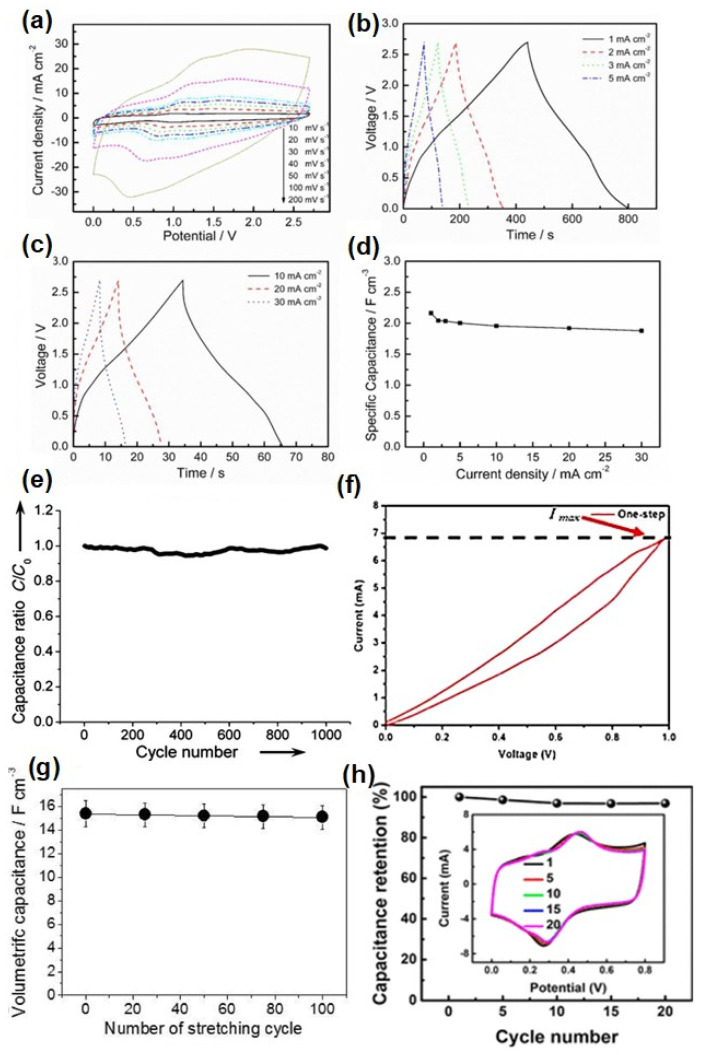
Electrochemical performance of ACM/MWCNTs@PDAA films with various polymerization charge densities measured in three-electrode mode in 1 M Et4NBF4–AN electrolytes. (**a**) CV curves at different scanning rates, (**b**,**c**) GCD curves at different current densities, (**d**) specific capacitances versus current density [45], (**e**) change in capacitance of stretchable fiber supercapacitor under stretching cycles [51], (**f**) C-V curve of graphene oxide/natural rubber latex nanocomposites-based supercapacitor [52], (**g**) change in capacitance of CNT-deposited rubber nanofiber electrode-based supercapacitor as a function of stretching cycles [53] and (**h**) capacitance values of isotropic buckled CNT film-based supercapacitor after cyclic stretching [54]. Reproduced with permission from ref. [49,51,52,53,54].

Wang et al. reported stretchable electrodes made up with rubber (ACM)/multiwall CNTs (MWCNTs) composite film using CPs poly(1,5-diaminoanthraquinone (PDAA) and polyaniline (PANI) through electro polymerization (Figure 5A) [49]. Among these materials, ACM (stretchable matrix) enables electrodes to be stretchable while enhancing the affinity to organic electrolytes [49]. MWCNTs provide effective conductive paths and facilitate the electro-deposition of CPs. The highest conductivity and good elastic resilience were achieved at the ACM/MWCNT film with 35 wt.% of MWCNTs [49]. The electrochemical performance of the stretchable electrode is improved by the electrodeposition of CPs, such as PANI and PDAA, onto the ACM/MWCNT films that supports pseudo-capacitance. The ACM/MWCNTs at PDAA anode and ACM/MWCNTs at PANI cathode exhibited high volumetric specific capacitances at a current density of 1 mA cm^−2^, which are 20.2 and 17.2 F cm^−3^, respectively (Figure 6a–d). The fabricated supercapacitor exhibits good pseudocapacitive behavior (Figure 6a) as well as has a fast charge–discharge characteristic and can be used as a replacement for batteries (Figure 6b,c). A high volumetric capacitance of 2.2 F/cm^3^ at 1 mA/cm^2^ are observed for the fabricated supercapacitor (Figure 6d). 

There were more examples of the rubber-based stretchable supercapacitor. In the work by Yang et al., a rubber fiber-based supercapacitor was introduced (Figure 5B) [51]. Multilayered coatings of CNT and electrolyte on rubber fiber produced a high stretchability of ~100% while retaining ~98% of capacitance after 1000 cycles (Figure 6e). Suriani et al. reported a graphene oxide/natural rubber latex nanocomposite-based supercapacitor [52]. They utilized an electrochemical exfoliation method to fabricate the composites as shown in Figure 5C. Their method achieved higher capacitance values of ~103.7 F/g than those fabricated with conventional production processes (Figure 6f). Yoon et al. also investigated rubber-based supercapacitors [53]. In their work, a single wall carbon nanotube (SWCNT) was coated on electrospun rubber nanofibers that was then used as a supercapacitor (Figure 5D). The supercapacitor using SWCNT/rubber fiber electrodes showed a stretchability of ~40% while showing a high volumetric capacitance of ~15.2 F/cm^3^ at 100 cycles of stretching (Figure 6g). Yu et al. also demonstrated a stretchable supercapacitor using a rubber-based composite electrode [54]. CNT films were formed on a prestrained rubber substrate (Figure 5E) that showed a stretchability of ~200%. The capacitance was not significantly changed after 20 cycles of stretching (Figure 6h).

## 4. Summary

The development of supercapacitors with high stability and performance has attracted significant interest in recent years. Stretchable supercapacitors used in wearable and stretchable electronics must endure various deformations, which include folding, twisting, and stretching. Under a given strain, stretchable supercapacitors should not exhibit a decrease in their performance. The fabrication of stretchable electrodes is a key factor for achieving highly stretchable supercapacitors. Stretchable electrodes with intrinsic or increased stretchability can be fabricated by modifying their structure. Supercapacitors comprising elastic polymers and stretchable structures are still being developed and studied. However, some key issues need to be resolved. Furthermore, the energy density of stretchable supercapacitors should be improved further for practical applications. Moreover, in order to be applied in the field of wearable electronics, stretchable supercapacitors should withstand a greater deformation as well. 

## Data Availability

The datasets used and/or analyzed during the current study are available from the corresponding author on reasonable request.

## Abbreviations

ACM	Acrylic rubber
AN	Acetonitrile
ATR-FTIR	Attenuated total reflectance Fourier transform infrared
CNT	Carbon nanotube
CP	Conducting polymer
CV	Cyclic voltammetry
DETA	Diethylenetriamine
DMAc	Dimethylacetamide
EDLC	Electric double-layer capacitor
Et4NBF4	Tetraethylammonium tetrafluoroborate
FE-SEM	Field emission scanning electron microscopy
GCD	Galvanostatic charge discharge
MWCNT	Multiwall carbon nanotube
oASSC	Organic semiconducting single crystal
PDAA	Poly(1,5-diaminoanthraquinone)
PANI	Polyaniline
QPE	Quasi-solid polymer electrolyte
TGA	Thermogravimetric analysis
XPS	X-ray photoelectron spectroscopy
UV	Ultraviolet
